# Transcranial ultrasound in the critically ill patient: a narrative review

**DOI:** 10.1186/s40635-025-00787-z

**Published:** 2025-08-18

**Authors:** R. M. J. Cashmore, M. Czosnyka

**Affiliations:** 1 Department of Critical Care, King’s College Hospital, London, UK; 2 Division of Neurosurgery, Department of Clinical Neurosciences, University of Cambridge, Biomedical Campus, Cambridge, UK; 3 Institute of Electronic Systems, Warsaw University of Technology, Warsaw, Poland

**Keywords:** Doppler ultrasonography, Transcranial, Doppler ultrasound imaging, Critical care, Intensive care, Point-of-care diagnostics

## Abstract

Transcranial ultrasound is gaining widespread recognition as a useful bedside monitoring tool and non-invasive diagnostic device in the critically ill patient. The capabilities of transcranial ultrasound are themselves ever-increasing, and this, combined with improved physiological understanding, affords insights into pathophysiological processes often concealed from the bedside critical care clinician. Transcranial ultrasound remains unique in regard to its non-invasive, rapid, and critically composite blood flow velocity-centric (not pressure-centric) information. The mobility of transcranial ultrasound devices is of particular value to the largely immobile critically ill patient requiring multiple organ supportive therapies. In this review, we discuss some important origins of more modern composite techniques and highlight relevant major key concepts, whilst noting exciting frontier possibilities.

## Background

**Fig. 1:**
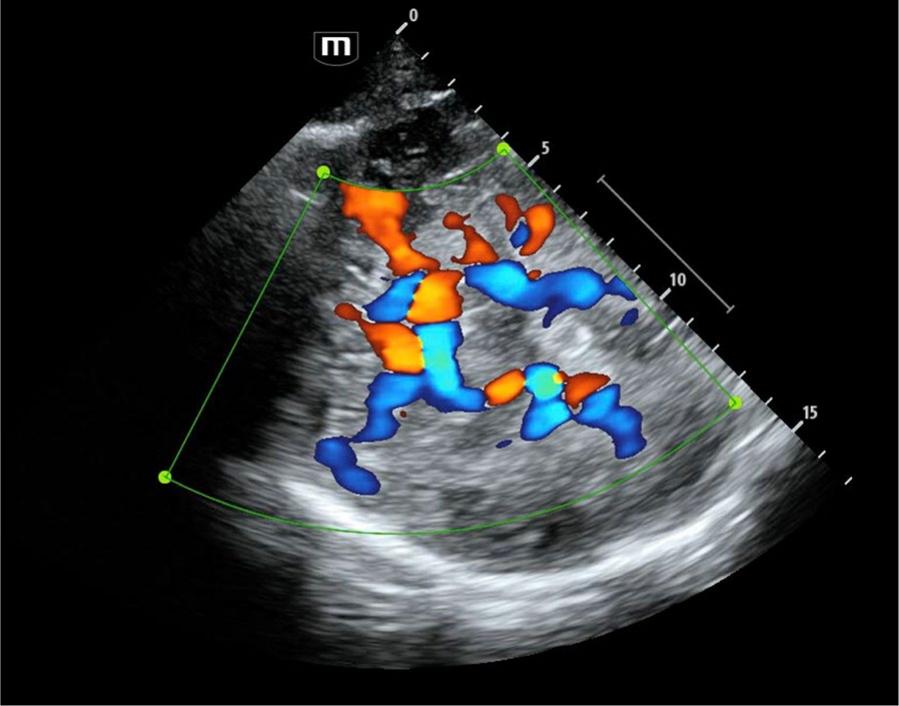
Cerebral vasculature demonstrated on TCCD in a patient who had previously undergone decompressive craniectomy for a traumatic brain injury. Image over-gained to demonstrate relative vessel positions

Transcranial B-mode imaging ultrasound was pioneered by Leksell [1] and further developed by Furuhata [2]. These techniques, combined with the renowned depth-ranged transcranial Doppler (TCD) technique first described by Aaslid [3], have led to the evolution of modern transcranial colour-coded duplex (TCCD; Fig. 1) and triplex (TCCT; Figs. 5 and 6) scanning, and opened new avenues for clinical application [4, 5]. Advanced electronic-processing within point-of-care ultrasound (POCUS) machines can now facilitate the rapid acquisition of time-averaged values, angle correction and calculation of derived haemodynamic parameters [6].

**Fig. 2:**
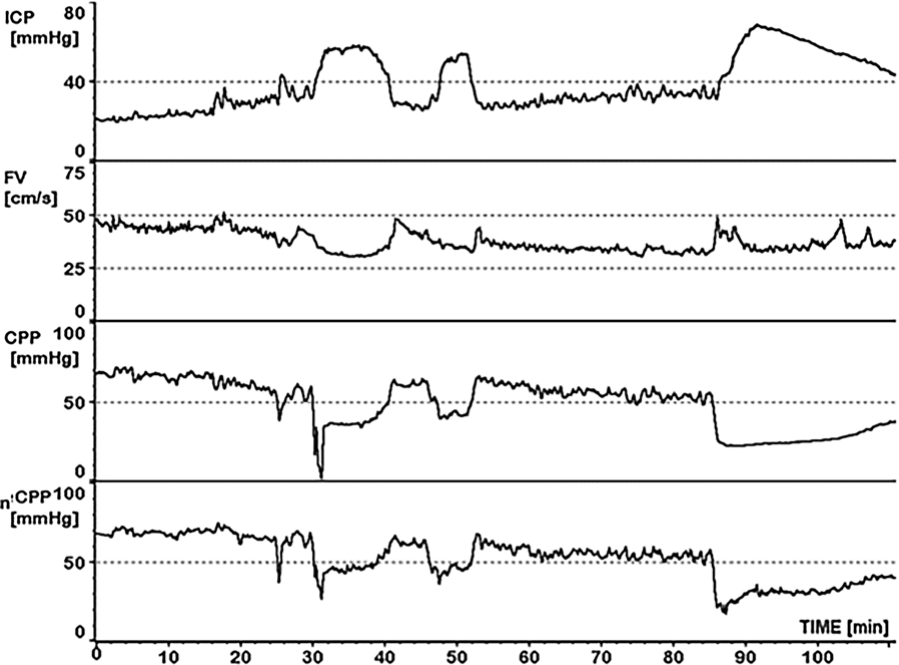
Run-time chart showing intracranial pressure (ICP), mean flow velocity (FV), invasively measured cerebral perfusion pressure (CPP) and non-invasively measured cerebral perfusion pressure (nCPP). The ability of nCPP to track CPP is clearly demonstrated over time

**Fig. 3:**
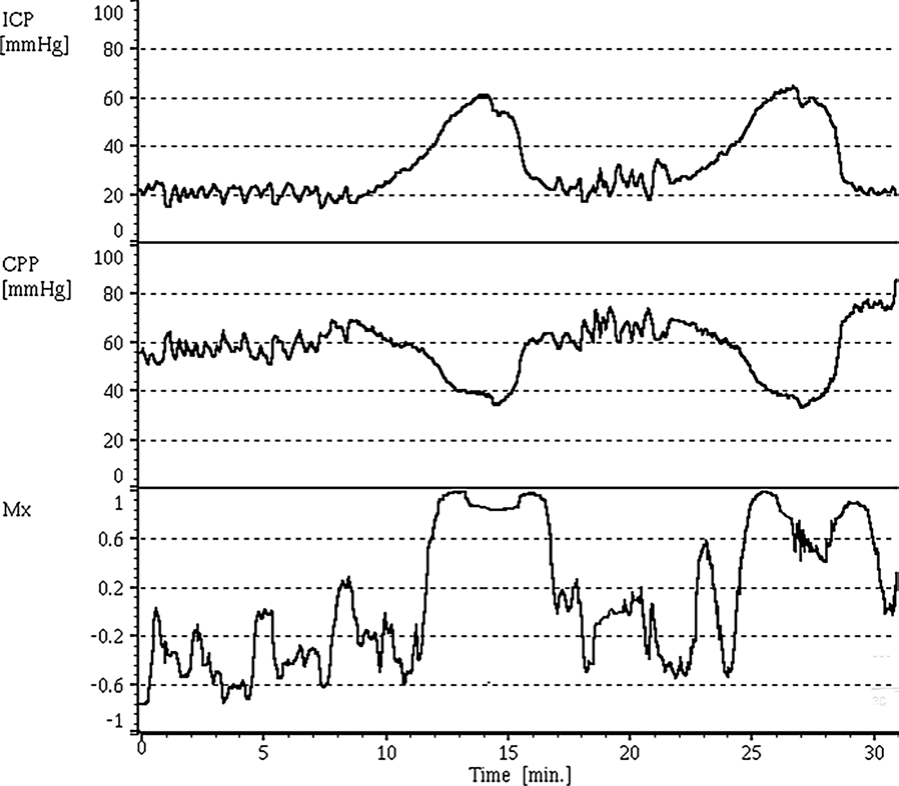
Simultaneously measured intracranial pressure (ICP), cerebral perfusion pressure (CPP), and the correlation coefficient Mx (CPP correlated with transcranial Doppler (TCD) measured mean flow velocity (FVm)) with each displayed against time. Above, ICP plateau-Lundberg A-waves- with corresponding periods of reduced CPP are shown. During these episodes, Mx becomes strongly positive, almost nearing 1. As such, during these spikes FVm is proportional to CPP, and autoregulation (where in health the correlation would be neutral or negative) can be seen non-functional

**Fig. 4:**
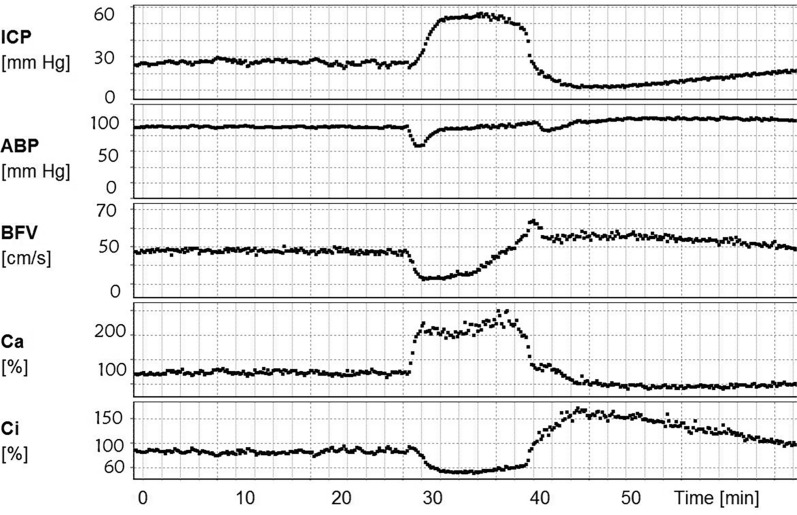
Run-time charts showing intracranial pressure (ICP), arterial blood pressure (ABP), blood flow velocity (BFV), high-pressure arterial compartment compliance (C_a_) and low-pressure venous and CSF compartment compliance (C_i_) plotted against time on the X-axis. A rise in ICP is provoked by cerebral vasodilatation, causing a plateau wave of ICP. The rise in ICP is synchronised with a decrease in Ci and arterial dilatation denoted by an increase in Ca

Extended-period ultrasound assessment via wearable head-set TCD devices permits application as non-invasive clinical monitors (Figs. 2, 3 and 4) [7, 8]. These devices can display real-time waveform data alongside other classically tracked bedside waveform parameters, and reveal increasing amounts of clinically important information. The wearable ultrasound devices are themselves becoming much less bulky and more refined, again with increasing computing power incorporated. Such devices are experimentally now reaching a point where they are able to measure cerebral blood flow (as opposed to velocity alone), which could in the future be used to trial novel clinical management strategies, or even herald a dramatic change in focus of clinical care [9]. Furthermore, extended period TCD can be incorporated into brain multimodal monitoring systems, potentially leading to more physiologically tailored care.

All these developments now mean that arguments against the widespread clinical application of transcranial ultrasound are largely outdated, and highlight the possibilities of transcranial ultrasound in modern critical care now and in the future.

### Critical care approaches

Adequate perfusion of oxygenated blood remains the principal goal of contemporary critical care. Historically, more readily measurable parameters such as pressure (arterial or intracranial) have been the bedside clinical measures used minute-to-minute to guide management [10]. Sound physiological and anatomical reasoning stemming from the early observations of the Monro–Kellie doctrine has developed into accepted intracranial pressure (ICP) targeted traumatic brain injury (TBI) management strategies [11–13]. The importance of controlling ICP below harmful levels is clear, and advanced invasive assessment methods have built on this [14, 15]. Whilst there has been variation in the practical methods of achieving the above, techniques have fundamentally been targeted at preserving flow through monitoring pressure parameters as available guides [12, 16]. Fundamentally, the goal to monitor and adjust flow and perfusion directly remains.

Advanced techniques to quantify blood flow have already been developed, and include phase-contrast magnetic resonance imaging (MRI) and positron emission tomography (PET). Whilst these techniques are indeed very informative, they are challenging to obtain in the critically unwell population, and remain fundamentally limited to providing only brief isolated snapshots in time of highly dynamic processes [17, 18]. As a direct consequence, this technology remains unsuitable to monitor dynamic pathophysiological changes, and unable to guide the inherent minute-to-minute critical care management interventions delivered.

Ultrasound technology, with its ability to measure beat-to-beat blood velocity as a minimum, offers the tangible bridge to achieving flow-centric care. The prior assumption of constant vessel diameter, and associated limitations of velocity not being proportional to flow, can potentially be overcome with transcranial ultrasound developments [9, 19]. TCCD is nearing a point to potentially facilitate measurement of vessel diameter, and combined with advanced techniques, makes flow and volume assessment more accurate, and absolute not relative changes an increasing reality [9, 19–21]. It is hoped these advances can improve accuracy and comparison data, whilst opening further avenues of study.

Whilst TCD has been established for assessment in isolated neurological diseases such as aneurysmal subarachnoid haemorrhage and large vessel occlusion (LVO) stroke, there is increasing recognition of the potential role it can play in broader critical care settings [7]. As the sequelae of delirium and post-intensive care syndrome become increasingly recognised, further investigation into the acute cerebrovascular changes of critical illness and its associated management strategies is expected to follow. Already the development and application of ultrasonographic contrast has allowed the ultrasonographic assessment of the cerebral microvasculature in a septic cohort [22]. Here, alterations in brain microcirculation were identified and associated with a poor outcome. These techniques are not only providing information previously unobtainable, but delivering it at the bedside.

The role of basic transcranial ultrasound in more general critically ill populations is also increasingly recognised and explored. Disease states typically managed outside a neuro-specific critical care such as hepatic encephalopathy or eclampsia are well suited to transcranial ultrasound application. These pathologies can additionally be complicated by coagulopathy or cerebral oedema, with the potential benefits of non-invasive monitoring and assessment clear. Of note, pulsatility index has been shown to increase in decompensated cirrhotic patients [23], and a recent study has demonstrated increased flow velocities and low resistance patterns in pregnant patients with eclampsia or severe pre-eclampsia [24]. Further understanding of disease phenotypes is expected to follow. Modern advances combined with the non-invasive nature and ability to perform extended-period or serial assessments without patient transfer are clear strengths.

### Transcranial doppler (TCD)

The Doppler waveform envelope describes the ultrasonographic pulse-waveform assessment, which can be highly informative to the bedside clinician. Originally described on the peripheral vasculature by Gosling and King, pulsatility index (PI) can provide information on vascular dynamics [25, 26]. PI, calculated by systolic flow velocity (FVs) minus—diastolic flow velocity (FVd) divided by the mean flow velocity (FVm), is usually in the approximate range of 0.7–1.1 [27]. Deviations both above and below this can have a number of causes, with some key causes outlined below.

Relevant to the neurocritical care clinician, a low pulsatility index can be seen in cases where distal resistance is markedly reduced, such as in arterio-venous malformation (AVM), or when associated with reduced velocities distal to a tight stenotic lesion [28].

A high pulsatility index (PI > 1.3) in the confirmed absence of hypocapnia or arterial hypotension may inform of inadequate cerebral perfusion pressure (CPP), and is probably most relevant to the critical care clinician [26, 29]. A globally or bilaterally raised pulsatility index in intracranial vessels can signal a raised ICP (again with care for additional confounders such as arterial hypotension, hypocapnoea, or intracranial atherosclerotic disease (ICAD) etc.). To this end, the potentially detrimental perfusion effects of hypocapnoea and hypotension can again be clearly illustrated.

Given the nature of the intracranial waveform under examination, in global pathology such as intracranial hypertension, it critically remains a sign of inadequate CPP, with the ICP itself proving fundamentally relative. What is most problematic and evident is the impaired CPP (either through raised ICP or low mean arterial pressure [MAP]), with the TCD waveform demonstrating a composite of the net effects on the vessel intracranially. Earlier studies have indeed trialed and proposed ultrasonic Doppler assessment for early intracranial hypertension screening and management [30–34]. Within acute traumatic brain injury (TBI) and intracranial hypertension, transcranial Doppler waveform appearance can help indicate the flow-pressure net status.

In TBI, the B-ICONIC consensus has highlighted the potential pragmatic role of the TCD/TCCD as part of a non-invasive assessment for intracranial hypertension [35]. In relation to TCD/TCCD, B-ICONIC suggests a threshold PI of 1.3 or more in conjunction with FVd < 20cm/s for consideration of cerebral blood flow changes potentially associated with a high ICP. B-ICONIC recommends that transcranial ultrasound, in addition to other non-invasive modalities, can be used to optimise critical care supportive therapies, e.g. therapeutic tier adjustment as per SIBICC principles [13], such as cardiovascular manipulation, ventilatory adjustment, sedation strategies, osmotherapy or surgical intervention [13, 35]. Importantly, high-quality interventional therapeutic-linked transcranial ultrasound studies in the critically ill are lacking, and as such standardising therapeutic response is not possible, with optimisation relying on physiological principles and comprehensive assessment. Interventional TCD remains an area of much needed research. Ongoing appropriate use of invasive monitoring in severe TBI is highlighted in the consensus [35]. 

### Critical closing pressure and non-invasive CPP

In addition, with TCD-derived signals, it is possible (although with limited accuracy) for formal calculation of non-invasive ICP estimation and the more complex critical closing pressure [6]. Rising critical closing pressure (CrCP) is an index of raised ICP or increased cerebrovascular resistance. The difference between diastolic blood pressure and CrCP is a useful metric of the margin between current working state and disappearance of diastolic blood flow velocity, which is in turn a marker of ischaemia [36]. Increasing general intensive care neurological assessment and management will be an important clinical goal over the next decade.

Simple formula for non-invasive estimation of CPP has gained popularity in clinical research:
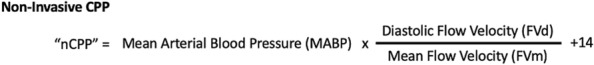


These unique nuances of the intracranial compartment in health and disease are suspected to be increasingly important [37]. Early work by Lindegaard explored the differential of hyperaemia or high blood flow and vasospasm [38]. Post-cardiac arrest patients with degrees of hypoxic–ischaemic injury, whilst a slightly pathologically unique brain-injury cohort, have been assessed using TCD [39]. Here, the identification of hyperaemic change and subsequent flow patterns may be clinically important. In the less frequent setting of laminar flow venous–arterial extra-corporal membrane oxygenation, where pulsatile waveforms can be lacking or minimal, TCD can still be informative, though with due care and consideration needed in interpretation [40].

### Autoregulation

The brain requires an adequate supply of oxygenated blood to survive, and ensuring overall adequacy of this requirement-delivery match is critical and carefully controlled in health within usual limits through various autoregulatory processes [41, 42]. Autoregulation has been studied for some time now, with the precise threshold of adequacy itself varying according to regional and global demand effects through cellular function and metabolism and pharmacological agents to name but a few [18]. In the pathological state of acute brain injury (ABI) this requirement-delivery matching capacity fails locally or globally, and ischaemia then infarction can ensue. Indeed, systemic haemodynamic monitoring, including that of cardiac output and systemic perfusion, has been explored as important factors in ABI care, recognising the limitations of blood pressure alone [43].

Calculated correlation coefficients for CPP versus TCD measured mean flow velocity (FVm) and systolic flow velocity (FVs), termed Mx and Sx, have been constructed facilitating assessment of dynamic autoregulation [44]. Static bedside tests such as transient carotid compression and breath holding can also be performed to assess autoregulation, but provide much less information than dynamic measures, and in the case of carotid compression are not to be undertaken without careful consideration at the very least [45, 46]. Analysis of Mx and Sx derived variables has shown their role in identifying patients with impaired autoregulation; itself associated with worse neurological outcomes. From this, an entirely non-invasive technique to calculate autoregulation status has been used by taking measurements of arterial blood pressure via a Finapress® and FVm by TCD, yielding the correlation coefficient termed ‘nMXa’ [47]. The non-invasive and dynamic nature of such a variable remains very appealing.

### Compartmental compliances of brain

Exploration of the cerebral pulsatile blood volume, and division by either pulsatile component of arterial blood pressure or intracranial pressure can also provide numerical values for the high-pressure arterial compartmental compliance (*C*_*a*_) and the low-pressure venous and CSF compliance (*C*_*i*_) [20, 21]. Here, with baseline fluctuations of volume naturally occurring through pulsatile cerebral blood flow, relative changes in compliance can be tracked over time. A correlation coefficient between *C*_*a*_ and *C*_*i*_ can again demonstrate periods of intact (negative correlation) or absent autoregulation (positive correlation). With these methods, the Monro–Kellie doctrine can be indirectly assessed in real-time [21].

Decreasing cerebrovascular time constant (Ca multiplied by cerebrovascular resistance) is clinically helpful to predict cerebral vasospasm in acute patients after subarachnoid haemorrhage [37]. The limitation of transcranial ultrasound to assess only larger and medium-sized intracranial vessels, though present, is an area of ongoing research, with work already underway into transcranial microvascular ultrasound imaging [22, 48]. Despite this progress, the complexities of end-oxygen delivery at mitochondrial level to neuronal tissue are increasingly recognised [49].

### Venous TCCD

**Fig. 5:**
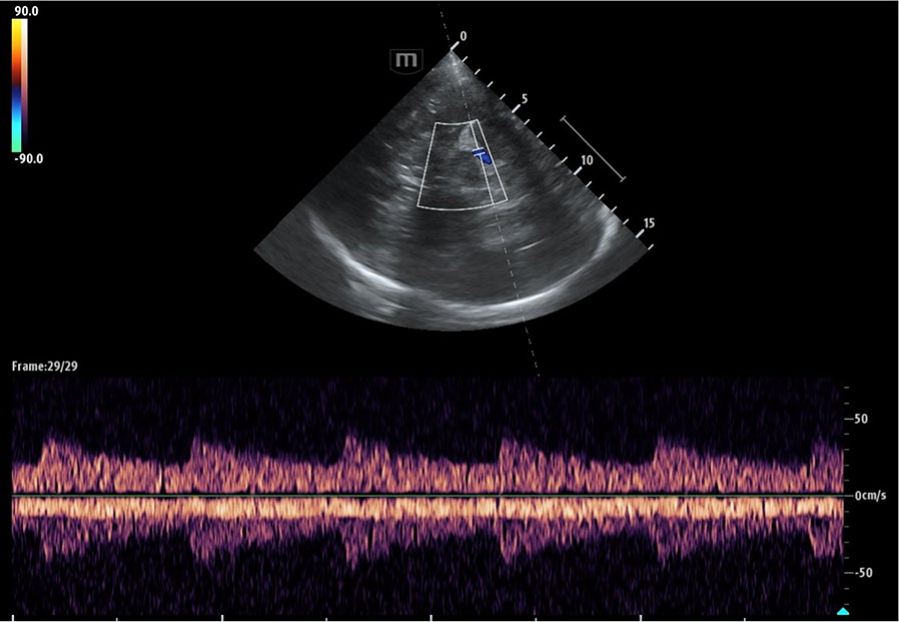
Transcranial colour-coded triplex and pulse-wave Doppler of the branching posterior cerebral artery with additional basal vein of Rosenthal waveform demonstrated within spectral Doppler trace

**Fig. 6:**
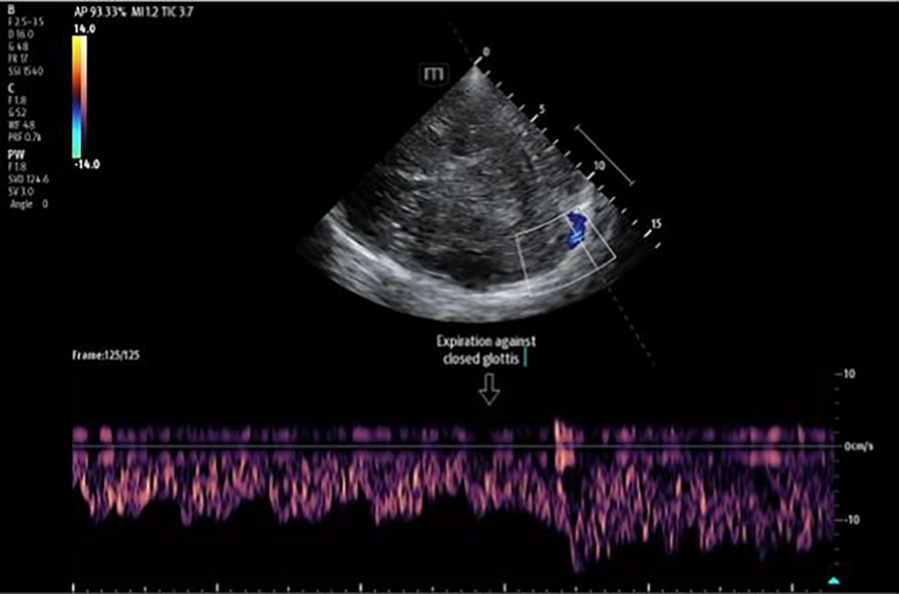
Transcranial colour-coded Triplex of the contralateral transverse sinus with respiratory influence demonstrated

Research within general intensive care continues after the elusive tissue perfusion goal. The complexities of fluid management, systemic filling pressures and venous or right-sided cardiac function factors are actively being explored [50]. Ultrasound techniques are frequently employed, and core to assessment and subsequent management [51]. Currently, neurocritical assessment and management of jugular venous outflow in acute situations is uncommon, and frequently limited to basic head-positioning and consideration of intra-thoracic pressures [52]. Despite this, intracranial venous TCCD is possible and allows increasingly holistic intracranial vascular assessment [53–55]. Assessments of blood flow in the basal veins and cerebral venous sinuses (Figs. 5 and 6) add important information to the other side of the circulation often neglected. In one study, velocity in the basal vein of Rosenthal and the straight sinus has shown, within a range, a relationship with ICP [56]. As known to clinicians during procedures such as central venous cannulation, further confirmed in a study involving healthy volunteers, there is a large influence of respiratory activity on blood flow at the cervical level [57]. Effective methods to measure and infer from arterial, venous, parenchymal, and cerebrospinal fluid biological signals continue to be sought to guide modern clinical care.

### Broader Doppler assessment application

Doppler ultrasound itself continues to be used in multiple areas of vascular assessment such as organ transplant, peripheral vascular disease, cardiothoracic and peri-operative care. Waveform analysis techniques employed in oesophageal Doppler cardiac output monitors demonstrate the informative data that can be obtained from waveform analysis [58]. Subsequent management techniques and protocols have followed. At present, whilst there is recognition and documentation of changing flow patterns in progressive states of cerebral hypoperfusion, waveform analysis and management protocols per se are not established [59, 60]. The TCD waveform morphology, itself a composite representation of local and systemic factors, provides unique information about net intracranial forces. The possibilities of further transcranial Doppler waveform analysis are suspected to hold important future insights.

TCD assessment of neurovascular coupling is possible, though predominantly the remit of research in non-critical care settings currently [61]. Whilst more limited in critically unwell patients, dynamic functional TCD assessments of neurovascular coupling, PCA flow velocities to light stimulus, etc., can with modification be applied to selected critically ill populations.

Transcranial tissue Doppler (TCTD) and velocimetry is a largely experimental application of transcranial ultrasound technology. TCTD assesses tissue displacement at multiple depths to provide information on tissue compliance. It will be interesting to monitor the development of TCTD technology for potential future roles in the critically unwell patient [62].

The diagnosis of Death by Neurological Criteria has been noted to have international variations, and is the focus of increasing scientific study and societal debate [63]. Transcranial Doppler has been proposed as one possible ancillary testing modality; however, its application is not uniform and multiple factors require careful consideration beyond the remit of this review [64].

### Structural TCCD

**Fig. 7:**
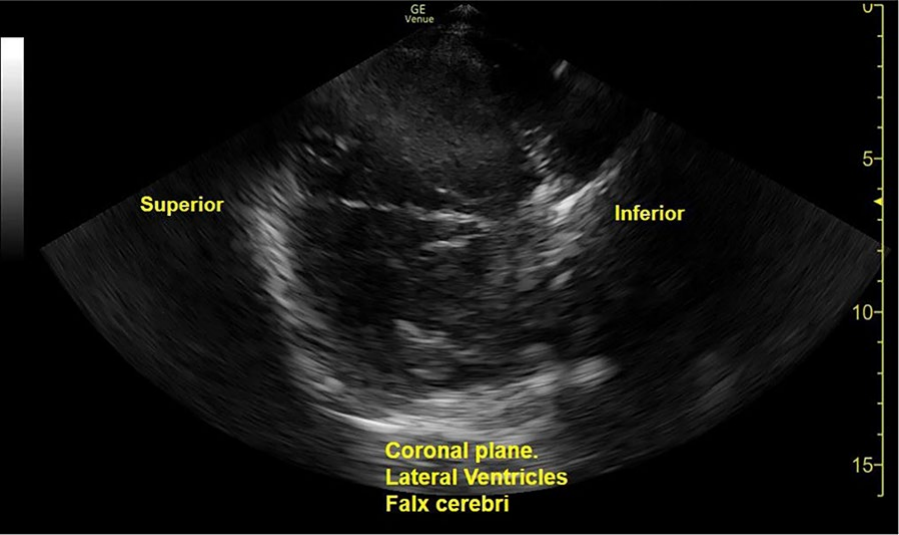
A Brightness-mode structural image in the coronal plane via the transtemporal window. The contralateral temporal bone is visualised along with the cerebral hemispheres, and the lateral ventricles in the centre of the image. The echogenic skull base bony structures inferior

**Fig. 8:**
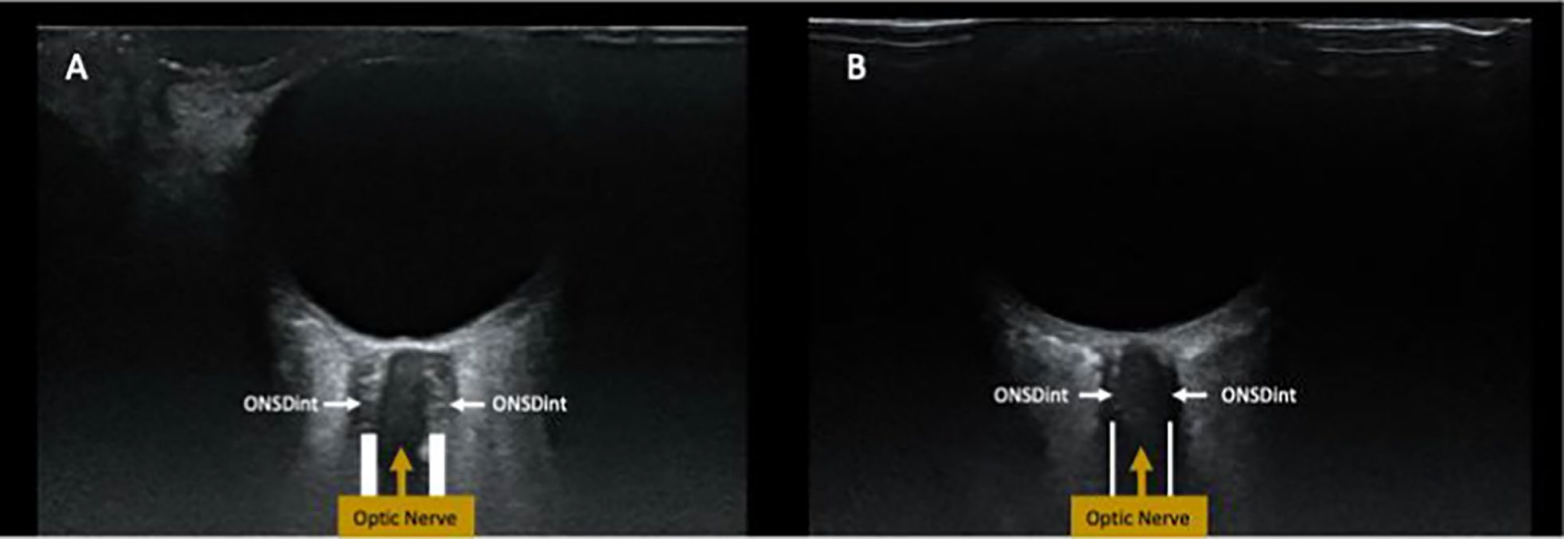
Optic nerve sheath ultrasound. Two examples of optic nerve sheath images: **A** in a patient with intracranial haemorrhage secondary to an arterio-venous malformation, **B** in a healthy individual. The ONSDint (as per Hirzallah et al [66]), is denoted by the outer border of the radiolucent parallel lines shown and annotated. ONSDexternal corresponds to the wider, outer borders, of the outer dark parallel lines (not annotated). 3 mm depth is not shown, but is important for performing correct diameter measurement

The capability of modern point-of-care machines to provide 2-dimensional structural images has opened new avenues for research (Figs. 7 and 8) [4, 5]. Structural assessment, whilst in its earlier stages, developing alongside technological advance, and to date most often assessed in the axial plane, holds much promise. Incorporation of structural images to TCD techniques improves the precision of vascular assessments, and increasingly allows for rapid parenchymal, extra-axial and ventricular assessments. Clearly, MRI and CT imaging will continue to play core roles in the management of acute neurological pathology, and accordingly, much caution is needed in the interpretation of structural ultrasound findings given limited transcranial windows for ultrasonographic assessment and frequent artefacts seen [5]. Fusion imaging, where a landmark-registered CT or MRI is used to overlay and guide transcranial ultrasound image navigation is also being explored [65].

### Optic nerve sheath diameter

Ultrasonographic assessment of optic nerve sheath diameter (ONSD) is an increasingly recognised examination technique that can also aid in the acute assessment of suspected raised intracranial pressure (Fig. 8). ONSD, whilst initially considered a relatively simple and rapid assessment technique, does have important nuance and technical challenge, with inaccurate measurement easily obtained in error. To aid the treating clinician, a recent consensus of structured assessment method (ONSD internal diameter specified) has been published [66]. The importance of ensuring safe thermal index (TI) ≤ 1 and mechanical index (MI) ≤ 0.23 in this technique [66], along with ALARA principle (applicable to all neuro-ultrasound) and meticulously careful practical performance, is very important to avoid injury. A study involving patients with acute brain injury concurrently undergoing invasive ICP monitoring demonstrated the potential value of combining ONSD measurement with venous straight sinus systolic flow velocity for identifying intracranial hypertension [67]. The B-ICONIC consensus statement has also highlighted the potential role of ONSD, noting an ONSD threshold of 6 mm, in addition to TCD/TCCD metrics when non-invasively assessing ICP [35]. B-ICONIC does again highlight the ongoing central role of invasive monitoring where suitable, whilst also combining different modalities and clinical correlation noted [35]. Some uncertainty remains as to the temporal resolution of increases and decreases in ONSD with ICP fluctuations and interobserver variability.

## Conclusion

Transcranial ultrasound represents an important and underutilised modality in the clinical management of critically unwell patients.

As an interval assessment tool for pathology or complication, or clinical monitor used to guide dynamic critical care, transcranial ultrasound provides unique and rapid information non-invasively at the bedside. Whilst no measure, tool or method will be adequate in isolation, we believe that transcranial ultrasound provides particularly valuable clinical information in the critically unwell patient. Further research is required on how best to incorporate the biological signals obtained from this technique into holistic critical care management strategies, with a physiological approach, extrapolated data and expert consensus currently bridging the evidence gap. How the clinician interprets and actions these signals, specifically in relation to interventional change, will be a key focus for clinical research in the coming years.

### Take-home message

Transcranial ultrasound is a stethoscope for the brain, offering a glimpse into the otherwise hidden black-box of cerebrovascular dynamics in the unconscious patient. As a point-of-care assessment tool or extended-period monitor, Transcranial ultrasound delivers valuable and unique physiological information to the bedside. Currently underutilised in the clinical management of critically unwell patients, transcranial ultrasound can be integrated into cohesive clinical management strategies and is a key focus for future research.

## Data Availability

There is no further availability of data or materials.
